# Transcriptomic similarities and differences in host response between SARS-CoV-2 and other viral infections

**DOI:** 10.1016/j.isci.2020.101947

**Published:** 2020-12-16

**Authors:** Simone A. Thair, Yudong D. He, Yehudit Hasin-Brumshtein, Suraj Sakaram, Rushika Pandya, Jiaying Toh, David Rawling, Melissa Remmel, Sabrina Coyle, George N. Dalekos, Ioannis Koutsodimitropoulos, Glykeria Vlachogianni, Eleni Gkeka, Eleni Karakike, Georgia Damoraki, Nikolaos Antonakos, Purvesh Khatri, Evangelos J. Giamarellos-Bourboulis, Timothy E. Sweeney

**Affiliations:** 1 Inflammatix, Inc., 863 Mitten Road, Suite 104, Burlingame, CA 94010, USA; 2 Institute for Immunity, Transplantation and Infection, School of Medicine, Stanford University, Palo Alto, CA 94305, USA; 3 Center for Biomedical Informatics Research, Department of Medicine, Stanford University, Stanford, CA 94305, USA; 4 Department of Internal Medicine, University of Thessaly, Larissa General Hospital, Greece; 5 Intensive Care Unit, Latseion General Hospital of Elefsis, Greece; 6 Intensive Care Unit, Aghios Dimitrios Thessaloniki General Hospital, Greece; 7 Intensive Care Unit, AHEPA Thessaloniki General Hospital, Greece; 8 4th Department of Internal Medicine, National and Kapodistrian University of Athens, Medical School, 124 62 Athens, Greece

**Keywords:** Molecular Biology, Immunology: Bioinformatics, Transcriptomics

## Abstract

The pandemic 2019 novel coronavirus disease (COVID-19) shares certain clinical characteristics with other acute viral infections. We studied the whole-blood transcriptomic host response to severe acute respiratory syndrome coronavirus 2 (SARS-CoV-2) using RNAseq from 24 healthy controls and 62 prospectively enrolled patients with COVID-19. We then compared these data to non-COVID-19 viral infections, curated from 23 independent studies profiling 1,855 blood samples covering six viruses (influenza, respiratory syncytial virus (RSV), human rhinovirus (HRV), severe acute respiratory syndrome coronavirus 1 (SARS-CoV-1), Ebola, dengue). We show gene expression changes in COVID-19 versus non-COVID-19 viral infections are highly correlated (r = 0.74, p < 0.001). However, we also found 416 genes specific to COVID-19. Inspection of top genes revealed dynamic immune evasion and counter host responses specific to COVID-19. Statistical deconvolution of cell proportions maps many cell type proportions concordantly shifting. Discordantly increased in COVID-19 were CD56^bright^ natural killer cells and M2 macrophages. The concordant and discordant responses mapped out here provide a window to explore the pathophysiology of the host response to SARS-CoV-2.

## Highlights

• Whole blood transcriptomics were generated via RNAseq for 62 COVID-19 patients
• Curated 23 whole blood transcriptomic studies (1855 samples) of non-COVID-19 viral infections
• Discovered 416 COVID-19-specific genes, despite overall correlation with non-COVID-19
• Revealed subset of immune cell proportions discordantly shifted in COVID-19 infections

## Graphical Abstract

**Figure:**
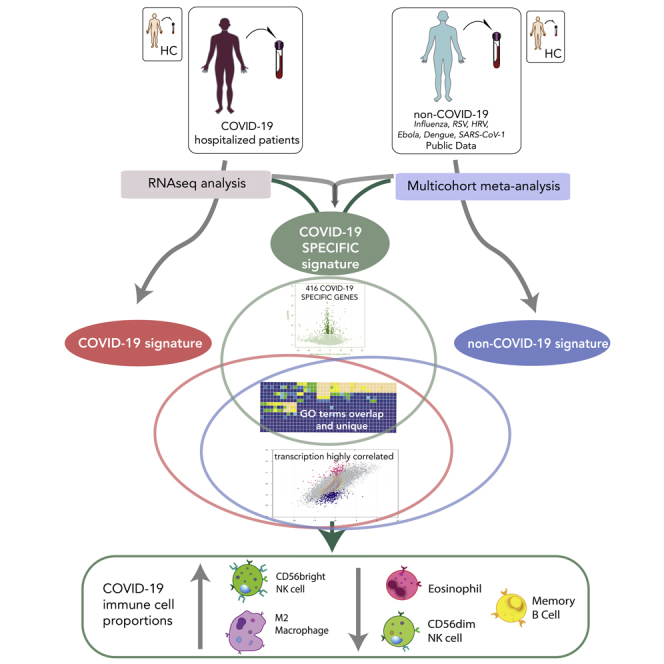

## Introduction

A novel coronavirus, severe acute respiratory syndrome coronavirus 2 (SARS-CoV-2), has developed into a global pandemic, resulting in more than 47.9 million cases and 1,221871 deaths across 235 countries as we write (WHO, accessed 5 Nov 2020) (Zhou et al., 2020). Contextually, this pandemic has surpassed the severe acute respiratory syndrome coronavirus 1 (SARS-CoV-1) 2003 pandemic by almost 6000-fold in total cases whereby SARS-CoV-1 resulted in 8,098 cases, took 12 months to contain, and had a 9.6% mortality rate (World Health Organization (WHO) accessed 1 Jun 2020). The novel SARS-CoV-2 virus, the causative agent for 2019 novel coronavirus disease (COVID-19), is highly communicable and despite urgent and resource-intensive efforts globally, we have no proven vaccine or efficacious treatment available (Callaway, 2020).

Early in a pandemic, it is imperative to understand what is similar in the host response to the novel virus when compared to other known viruses in order to rapidly rule in or rule out recyclable treatments and/or vaccination strategies. At the same time, it is also critical to understand the differences in this disease in order to search for novel therapeutics. The human immune system has evolved over millions of years to protect the host from microbes (Medzhitov, 2007; Longo et al., 2015). Understanding the overlap, or lack thereof for the most basic immunological features such as the virus's ability to inhibit the interferon response or to infect host cells with an antibody-dependent infection enhancement, can drive medicine rapidly in a life-saving direction (Jaume et al., 2012; Wang et al., 2016; Mesev et al., 2019; Blanco-Melo et al., 2020). In the last decade alone, we have already responded to pandemics of H1N1, chikungunya, Zika, and near-pandemics of two other coronaviruses, SARS-CoV-1 and Middle East respiratory syndrome-related coronavirus (MERS), from which valuable insights can be applied (Morens and Fauci, 2020). COVID-19 clearly shares immunological features with other viral responses, such as interferon activation, simultaneous repression of immune cells, and changes in metabolism including glucose and iron regulation as shown by cytokine and cytometry studies (Drakesmith and Prentice, 2008; Blanco-Melo et al., 2020; Catanzaro et al., 2020; Wilson et al., 2020). Notable features of COVID-19 include high rates of acute respiratory distress requiring mechanical ventilation; clinical coagulopathy; features of a cytokine storm and/or viral sepsis, and a high case fatality rate (Tay et al., 2020).

The COVID-19 pandemic has resulted in the halt of normal life across the globe in an attempt to slow the spread of the virus. Computational methods leveraging data generated prior to the pandemic present an advantage to push forward the aforementioned knowledge discovery. Studies comparing COVID-19 to healthy controls (HCs) are useful; however, they do not explain the similarities and differences seen in the COVID-19 syndrome versus other viral infections; hence, we have leveraged our multicohort, conormalization method to execute a head-to-head comparison of COVID-19 to non-COVID-19 viral infections.

Our approach involves a multi-cohort analysis of transcriptomic host response data to investigate host inflammation. The core discovery method leverages biological, clinical, and technical heterogeneity across data sets to identify generalizable disease biomarkers. We have repeatedly demonstrated that host response can be a generalizable sensitive and specific diagnostic and prognostic marker for presence, type, and severity of infections (Sweeney et al., 2015, 2016b, 2018a), of note viral infections (Andres-Terre et al., 2015) but also in autoimmune diseases, vaccination, tuberculosis, cancer, and organ transplant (Li et al., 2012; Khatri et al., 2013; Chen et al., 2014; Andres-Terre et al., 2015; Sweeney et al., 2015, 2016a, 2016b, 2018a, 2018b; Sweeney and Khatri, 2015; Warsinske et al., 2018a, 2018b; Haynes et al., 2020; Mayhew et al., 2020). We have shown in methodological work that this method produces results with the greatest reproducibility in independent cohorts (Sweeney et al., 2017).

In this work, we used RNAseq to profile whole blood samples from 62 patients with COVID-19 prospectively enrolled in Athens, Greece, together with 24 HCs. We simultaneously compiled a database of clinical viral infections from 23 studies of >1,800 samples to represent the conserved immune response to a broad range of viral infections including influenza, respiratory syncytial virus (RSV), human rhinovirus (HRV), SARS-CoV-1, Ebola, and dengue. We here report on the results of a comparison of host responses to SARS-CoV-2 and other viruses. We mapped out their similarities and differences at the gene level, pathway level, and cell proportion level, as a first step to gain a better understanding of this novel pandemic virus and demonstrate that a large portion of the response is in fact similar to previous viral infections. This is immensely valuable as it demonstrates that it is this conserved host response that allows for pandemic preparedness and response. Our implementation of computational methods comparing SARS-CoV-2 to known circulating viruses yields a COVID-19-specific gene signature for differentiating the host response, which warrants further investigation.

## Results

### Differential expression analysis of transcriptome profiles of patients with COVID-19

**Figure 1:**
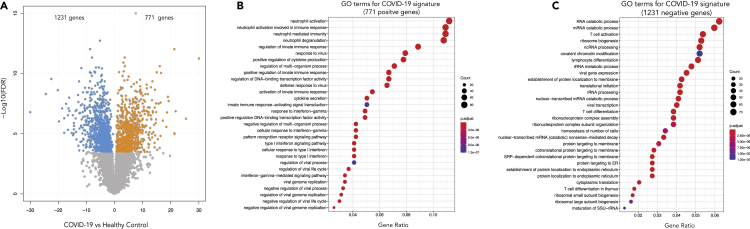
RNA-seq data for patients with COVID-19 versus healthy control and pathway analysis of the COVID-19 signature (A–C) (A) Significance score [defined as -log10(FDR)] versus mean difference of co-normalized log2-transformed expression data between patients with COVID-19 (n = 62) and healthy controls (n = 24). The chosen cutoff of ES ≥ 1 or ≤ −1 with FDR ≤0.05% yields the 2,002 COVID-19 signatures, including 771 positively regulated genes and 1,231 negatively regulated genes. GO term enrichment analysis of positive (B) and negative (C) gene sets reveals increased neutrophil function enrichment and decreased T-cell-related pathways (gene ratios represent the number of genes in our gene set within that pathway). The gene ratio (x axis) is the ratio of the number of genes in our data enriched in a given gene set (pathway) to the total number of genes in that pathway.

We prospectively enrolled and sequenced RNAseq from whole blood from 62 patients with COVID-19 and 24 HCs (Table 1). Differential expression analysis of 86 peripheral blood samples identified 2,002 differentially expressed genes (771 over-expressed, 1,231 under-expressed; Figure 1A, Table S2A) with absolute Hedges' g effect size (ES) which is the difference between groups as a proportion of variability in the groups (Hedges' g ES) ≥ 1 and false discovery rate (FDR) ≤0.05%), referred to as the “COVID-19 signature”. We performed pathway enrichment analysis of the COVID-19 signature using Gene Ontology (GO) terms. The 30 most significant pathways for 771 over-expressed genes included neutrophil activation, innate immune response, immune response to viral infection, type-I interferon signaling, and cytokine production (Figure 1B) and for 1,231 under-expressed genes include lymphocyte differentiation and T-cell activation and regulation (Figure 1C). These results suggest that, in response to SARS-CoV-2 infection, T cells are suppressed, whereas neutrophils are activated as a hallmark of its overwhelming host response represented in the transcriptomic changes. High neutrophil-to-lymphocyte ratios have been observed as a marker of severity in sepsis, cancer, and pneumonia (Diao et al., 2020; Lagunas-Rangel, 2020; Liu et al., 2020; Qin et al., 2020).

**Table 1:** Baseline characteristics table for patients with COVID-19

Characteristic	Patients with COVID-19
N	62
Age in years: median [IQR] (n)	61 [52,70] (61)
Gender = male (%)	40 (65)
SOFA (sequential organ failure assessment) score	2 [1,4] (61)
APACHE II (Acute Physiology And Chronic Health Evaluation II)	6.5 [4,9] (56)
Pneumonia severity index	89.5 [65,104.5] (48)
White blood cell (mm3)	6180 [4910,8420] (59)
Neutrophils	75.5 [65.43,84.13] (59)
Lymphocytes	15.69 [10.5,22.55] (59)
Platelets (k/mm3)	195.2 [158.8, 238.8] (58)
Lactate (mmol/L)	1.55 [1.04,2.08] (30)
pO2.FiO2 (mmHg)	255.35 [112.5,310.8] (50)
Creatinine (mg/dL)	0.9 [0.7,1.015] (58)
PCT (procalcitonin) (ng/mL)	0.1 [0.04,0.41] (49)
CRP (C-reactive protein) (mg/L)	78.85 [29.48,175.8] (60)
Days between onset symptoms and sampling	6 [4,8] (53)
Days between intubation and sampling	1 [0.5, 1.5] (23)
Days between hospital admission and intubation	2 [1, 3.5] (23)

All continuous variables are reported as median and interquartile ranges (IQRs) (n).

### Identification of host response genes to viral infections through multi-cohort analysis

**Figure 2:**
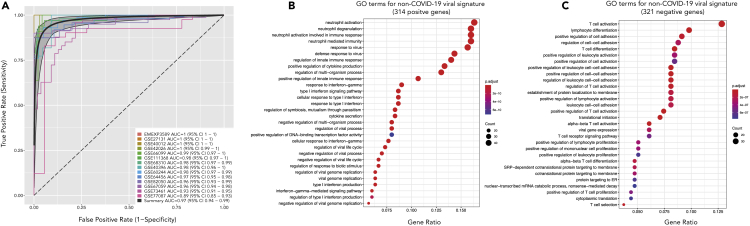
Metaintegration of 14 non-COVID-19 viral disease data sets and pathway analysis of non-COVID-19 signature genes (A–C) (A) ROC plots of the 635 non-COVID-19 viral signatures discovered using multicohort analysis with a cutoff of ES ≥ 1 or ≤ −1 and FDR ≤0.05% resulting in 314 positively regulated genes and 321 negatively regulated genes then plotted individually for each of the 14 data sets of viral infections (n = 652) and healthy controls (n = 672) identified. The consistent and high AUC values indicate that the signature is representative of all data sets, thereby embracing the heterogeneity which will increase generalizability. GO term enrichment analysis of positive (B) and negative (C) gene sets reveals increased neutrophil function enrichment and decreased T-cell-related pathways, similar to those in Figure 1 (gene ratios represent the number of genes in our gene set within that pathway).

Based on our previous results (Andres-Terre et al., 2015), we hypothesized that there is a conserved immune response to respiratory viral infections irrespective of age and genetic background of a patient or a virus. We identified 23 studies of acute viral infection and from these selected 14 as our discovery set for a non-COVID-19 viral signature (Table 2) and 9 were held out for validation. Statistical power analysis (Hedges and Pigott, 2001) found that even with high inter-study heterogeneity, we had more than 80% statistical power at p value = 0.01 for detecting absolute Hedges' g ES > 0.43 in these data sets (Figure S2). The multi-cohort analysis of 1,324 transcriptome profiles (652 patients with non-COVID-19 viral infections, 672 HCs) from these 14 studies using MetaIntegrator (Haynes et al., 2017) identified 635 differentially expressed genes (314 over-expressed, 321 under-expressed). The area under the curve (AUC) of a receiver operator characteristics (ROC) curve represents the discriminatory ability of the score to correctly identify true positive and/or true negatives. The closer to 1 the value is, the better the performance of the test, for example, a test that can discriminate if a patient or sample is virally infected or healthy. ROC plots for all of the discovery data sets using this signature illustrate the high sensitivity and specificity this gene list possesses, indicating genes that are highly discriminatory and hence likely to represent this conserved signature (Figure 2A, Table S2A). We refer to these 635 genes in short as the “non-COVID-19 viral signature”. Similar to the COVID-19 signature, GO analysis of over- and under-expressed genes in the non-COVID-19 viral signature identified a similar set of pathways highlighted by neutrophil and T-cell activation, respectively (Figures 2B and 2C).

**Table 2:** 14 Data sets used for discovery of the non-COVID-19 viral immune response

Accession	Platform	First author	PMID	Timing of diagnosis	Disease	Total sample number	N healthy controls	N viral	Age
GSE60244	GPL10558	Suarez NM	25637350	Within 24 hr of admission	Respiratory viral infection	111	40	71	Adults
GSE40012	GPL6947	Parnell GP	22898401	On admission to intensive care unit (ICU)	H1N1 influenza A	24	18	8	Adults
GSE40396	GPL10558	Hu X	23858444	On hospitalization	Febrile children with viral infection	44	22	22	Infants
GSE64456	GPL10558	Mahajan P	27552618	On hospitalization	Febrile children with viral infection	130	19	111	Infants
GSE42026	GPL6947	Herberg JA	23901082	On hospitalization	H1N1, RSV	74	33	41	Children
GSE67059	GPL6947	Heinonen S	26571305	Within 48 hr of admission to emergency department(ED)	HRV +/− symptoms	101	21	80	Infants
EMEXP3589	GPL10332	Almansa R	22852767	Within 24 hr of admission to ICU	Infected chronic obstructive pulmonary disease (COPD) in ICU with viral infections	9	4	5	Adults
GSE82050	GPL21185	Tang BM	28619954	Within 24 hr of admission	Influenza	39	15	24	Adults
GSE68310	GPL10558	Zhai Y	26070066	Within 48 hr of acute respiratory infection onset	Influenza and other respiratory viral infections	347	243	104	Adults
GSE73461	GPL10558	Wright VJ	30083721	On presentation of symptoms	Viral infection	149	55	94	Children
GSE111368	GPL10558	Dunning J	29777224	Within 24 hr of admission	Seasonal flu study, acute timepoints	163	130	33	Adults
GSE77087	GPL10558	de Steenhuijsen Piters WA	27135599	Within 24 hr of hospitalization	RSV	59	18	41	Infants
GSE66099	GPL570	Alder MN; Sweeney TE	27635771; 25972003	Admission to ICU	Viral infection	58	47	11	Children
GSE27131	GPL6244	Berdal J	21781987	On hospitalization	Severe flu A	14	7	7	Adult
Total						1324	672	652

### Validation of host response genes to viral infections in multiple independent data sets

**Figure 3:**
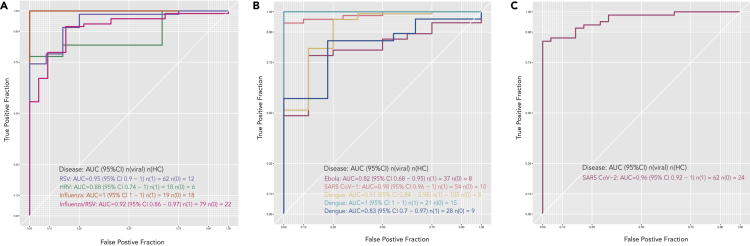
Validation of a global host immune response to viral infections (A) ROC performance of 635 non-COVID-19 signatures in 4 independent respiratory viral infection data sets including HRV, RSV, picornavirus, and influenza. (B) ROC performance in 5 additional cohorts of other viral infections to illustrate that this signature is broadly applicable to many viruses [Ebola (GSE122692), SARS CoV-1 (GSE5972), and dengue (GSE38246, EMTAB3162, GSE51808)]. (C) The signature is also tested in the 62 patients with COVID-19 and 24 HCs.

Next, we confirmed that the non-COVID-19 viral signature is conserved across viruses by validating it in several independent data sets. We calculate the non-COVID-19 viral score for a sample as the difference in geometric means of over-expressed and under-expressed genes. In four independent studies consisting of 236 samples (178 viral infections, 58 HCs; Table 3), the score accurately distinguished patients with a respiratory viral infection (influenza, HRV, or RSV) from HCs (Figure 3A). Second, we investigated whether the non-COVID-19 viral signature is observed in other severe viral infections including Ebola, dengue, and SARS-CoV-1 in five independent studies (50 HCs, 54 SARS-CoV-1, 37 Ebola, 154 dengue). In each study, the non-COVID-19 viral score also distinguished patients with a viral infection from HCs with high accuracy (Figure 3B). Third, we tested whether the non-COVID-19 viral signature would also distinguish patients with COVID-19 from HCs. We calculated the non-COVID-19 viral score for each of 62 patients with COVID-19 together with 24 HCs using the conormalized expression data. We found that non-COVID-19 viral score separated patients with COVID-19 from HCs with an AUC of 0.96 (Figure 3C), similar to SARS-CoV-1 (AUC = 0.98).

**Table 3:** Data sets for validation of the non-COVID-19 viral versus healthy signature

Accession	Platform	First author	PMID	Timing of diagnosis	Disease	Total sample number	N healthy controls	N viral	Age
GSE117827	GPL23126	Yu J	30339221	Within 24 hr of hospitalization	HRV	24	6	18	Children
GSE20346	GPL6947	Parnell G	21408152	At peak symptoms	Influenza	37	18	19	Unknown
GSE34205	GPL570	Ioannidis I	22398282	Within 42–72 hr of hospitalization	Influenza/RSV	101	22	79	Infants
GSE103842	GPL10558	Rodriguez-Fernandez R	29045741	Within 24 hr of hospitalization	RSV	74	12	62	Infants
Total						236	58	178	
GSE5972	GPL4387	Cameron MJ	17537853	Within 24 hr of hospitalization	SARS (CoV1)	64	10	54	Adults
GSE122692	GPL16686	Reynard S	30626757	Within 24 hr of hospitalization	Ebola	45	8	37	Adults
EMTAB3162	GPL570	van de Weg CA	25768297	On admission	Dengue	36	15	21	Adults and children
GSE51808	GPL13158	Kwissa M	24981333	On admission	Dengue	37	9	28	Adults and children
GSE38246	GPL15615	Popper SJ	23285306	Within 24 h of hospitalization	Dengue	113	8	105	Children
Total						295	50	245	

^a^indicates data sets not eligible for COCONUT

### Comparison of COVID-19 profile with non-COVID-19 viral infection profile

**Figure 4:**
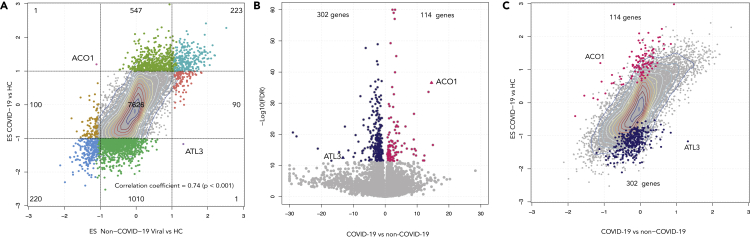
Comparison of COVID-19 signature with non-COVID-19 signature (A) Scatterplot of effect size for all 9,818 genes commonly present in all data sets between non-COVID-19 vs HC (x axis) and COVID-19 vs HC (y axis). Two thousand two COVID-19 signature genes from Figures 1 and 635 non-COVID-19 signature genes from Figure 2 are overlayed and colored, each of the 9 quadrants have a different color to allow for easy visualization of the overlap of Hedges' g ES from each signature. For example, teal in the top right quadrant are the genes that have an Hedges' g ES ≥ 1 for both the 2,002 COVID-19 signature genes and the 635 non-COVID-19 signature genes. Concordant host response between COVID-19 and other viral infections is reflected by 223 commonly positively (teal, top right) and 220 negatively (blue, bottom left) regulated genes in both. Discordant response is only seen in ACO1 whose expression is positively regulated in COVID-19 but negatively regulated in non-COVID and in ATL3 whose expression is negatively regulated in COVID-19 but positively regulated in non-COVID-19. (B) Using COCONUT conormalized data combined with a head-to-head comparison of COVID-19 and non-COVID-19 viral infections using Hedges' g ES ≥ 1 or ≤ −1 with FDR ≤0.05% yields 416 COVID-19-specific signatures, including 114 positively regulated genes and 302 negatively regulated genes. Significance score [defined as -log10(FDR)] vs mean difference of co-normalized log2-transformed expression data between patients with COVID-19 (n = 62) vs other viral infections (n = 652). (C) To illustrate the overlap of (A) and (B), the 416 COVID-19-specific signature genes from head-to-head comparison in (B) are shown in the same scatterplot in (A).

Next, we investigated similarities and differences in host response to SARS-CoV-2 and other respiratory viruses by comparing change in expression with respect to HCs across 9,818 genes that were present across all data sets. When considering the entire transcriptome, there was high correlation (r = 0.74, p < 0.001) between change in expression in response to SARS-CoV-2 or other respiratory viruses (Hedges' ES from COVID-19 vs HC comparison is plotted against ES from non-COVID-19 vs HC comparison in Figure 4A). We visualized “2,002 COVID-19 signature genes” and “635 non-COVID-19 signature genes” in the same ES scatterplot by different colors to highlight their relationships (Figure 4A and Table S2A). We observe that 7,626 genes uncolored in the middle (gray, with higher density in the center shown by contours) out of 9,818 profiled (77.7%) are not in the signature genes in either COVID-19 or non-COVID-19 viral infections. Given the high correlation (r = 0.74), it is not surprising that 223 genes are concordantly over-expressed (Hedges' g ES ≥ 1, FDR ≤0.05%), as well as 220 genes concordantly under-expressed with (Hedges' g ES ≤ −1, FDR ≤0.05%). Of the remaining genes from the “non-COVID-19 signature”, there are 90 genes over-expressed and 100 genes under-expressed in non-COVID-19; however, these had ES between −1 and 1 in the distribution of the COVID-19 ESs. As well, of the remaining genes from the “COVID-19 signature”, there are 547 genes over-expressed and 1,010 genes under-expressed in COVID-19 that had ES between −1 and 1 in the distribution of the non-COVID-19 ESs. We only found two genes that were completely discordant, thus completely oppositely regulated in COVID-19 and non-COVID-19 viral infections: Aconitase1 (*ACO1*) is over-expressed in COVID-19 and under-expressed in non-COVID-19 viral infections and Atlastin GTPase 3 (*ATL3*) is over-expressed in non-COVID-19 viral infections and under-expressed in COVID-19. Interestingly, *ACO1* is involved in iron metabolism, and heme appears to be interlinked with COVID-19 pathophysiology (Hopp et al., 2020). *ATL3* is required for endoplasmic reticulum (ER) membrane junctions and may be linked to viral replication sites (Monel et al., 2019).

Therefore, in order to identify a statistically significant set of genes differentially expressed in patients with COVID-19 compared to those with other viral infections, we employed COCONUT to conormalize the two disease types into a single matrix for comparison of 62 patients with COVID-19 versus 652 patients with non-COVID-19 viral infection. Conormalization with COCONUT allows for pooling of data across data sets while simultaneously removing batch-to-batch technical variance in a bias-free manner (Sweeney et al., 2016b). At Hedges' g | ES| ≥ 1 with FDR ≤0.05%, we found 416 genes we refer to as the “COVID-19-specific gene signature”, 114 over-expressed and 302 under-expressed in patients with COVID-19 than in those with non-COVID-19 viral infection (Figures 4B, Tables S2A and S2B). To illustrate the gain in identification of genes to investigate and re-iterate the value in this statistical method, this set of genes from (b) is highlighted in the same scatterplot from panel a (Figure 4C).

Unlike the “COVID-19 and non-COVID-19 viral signatures”, the pathway analysis of this gene set did not identify any statistically significant GO terms, potentially indicating novel pathophysiology unique to COVID-19. This combination of genes may include those less well annotated within pathways and thus less likely to result in statistically significance assignment to a pathway. Nonetheless, top ranked but statistically insignificant GO terms include muscle contraction, regulation of epithelial cell proliferation, and biological processes involved in lung and respiratory development for 114 positive genes, as well as pathways related to T-cell homeostasis and T-cell differentiation for 302 negative genes. The significance of these pathways in connection with clinical manifestation needs to be investigated further.

### Similarities and differences in pathways between COVID-19 and non-COVID-19 viral infection

**Figure 5:**
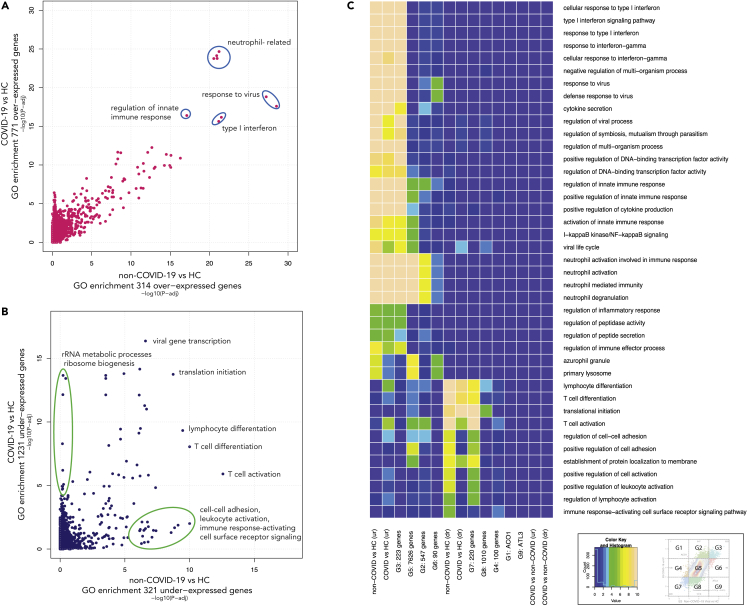
Summary of pathway analysis results Scatterplots of the significance level from pathway enrichment analysis between COVID-19 and non-COVID-19 viral infections obtained for positive genes in (A) and negative genes in (B), respectively. Significance is defined as -log10(BH-corrected p value) for each pathway. The concordance is seen in results for up-regulated genes between COVID-19 and non-COVID-19, while a degree of discordance is evident in down-regulated genes between COVID-19 and non-COVID-19. (C) Heatmap summary of pathway enrichment analysis for 15 gene sets of interest including COVID-19 vs HC (+) and (−), non-COVID-19 viral vs HC (+) and (−), COVID-19 vs non-COVID-19 viral (+) and (−), as well as gene lists from 9 groups segmented in Figure 4A as labeled in the legend key box. Values between 1 and 10 of -log10(BH-corrected p value) are plotted. ur, up-regulated; dr, down-regulated.

We expanded our comparison of significant pathways in response to SARS-CoV-2 versus non-COVID-19 viruses by including all pathways instead of only 30 most significant pathways. We found pathways for over-expressed genes are highly concordant between patients with COVID-19 and non-COVID-19 viral infections (Figure 5A), pathways for under-expressed genes are discordant (Figure 5B).

To amalgamate these findings, we performed hierarchical clustering of all pathway analysis results of all gene sets of interest including “three signature sets”: (1) COVID-19 vs HC (771 over- and 1,231 under-expressed), (2) non-COVID-19 viral vs HC (314 over- and 321 under-expressed), and (3) COVID-19 vs non-COVID-19 viral (114 over- and 302 under-expressed), as well as gene lists from the 9 groups by quadrant in Figure 4A (Figure 5C, Table S2A). To check the dependency of GO term enrichment results on the cutoffs for selecting signature genes, we tested three additional cutoffs (less or more stringent than the chosen one) each for COVID-19 vs HC, non-COVID-19 vs HC, or COVID-19 vs non-COVID-19 comparison. The results for over-expressed, under-expressed, and all genes from each cutoff together with the 9 gene sets from Figure 4A show a merging and comprehensive picture of pathway analysis results (Table S3, Figure S3), allowing one to focus on pathways of interest, either commonly significant across gene sets or uniquely significant in a gene set or a combination of genes of interest.

### Similarities and differences in changes in immune cell proportions between COVID-19 and non-COVID-19 viral infection

**Figure 6:**
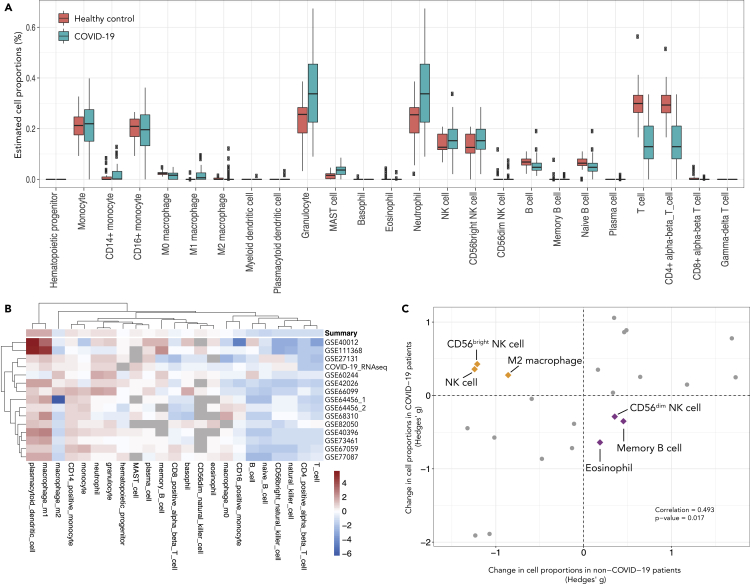
Statistical deconvolution of bulk transcriptome profiles using immunoStates of COVID-19 versus non-COVID-19 viral infections (A) Changes in cell proportions when comparing patients with COVID-19 to healthy controls. Note the trends of increased neutrophil and decreased T-cell proportions (median and interquartile range [IQR]). (B) Heatmap of changes in cell proportions of all data sets: non-COVID-19 and COVID-19. (C) Concordant and discordant changes in cellular proportions comparing COVID-19 to non-COVID-19 viral infections. Cell types that increased in COVID-19 (hence decreased in non-COVID-19) were CD56^bright^ NK cells, M2 macrophages, and total NK cells. Those that decreased in non-COVID-19 but increased in COVID-19 were CD56^dim^ NK cells, memory B cells, and eosinophils.

We estimated proportions of 25 immune cell types in bulk gene expression in blood samples from patients with COVID-19 or non-COVID-19 viral infections using immunoStates. In patients with COVID-19, we found immune cells from myeloid lineage (M1 macrophages, neutrophils, and MAST cells) increased significantly (FDR ≤10%) and lymphoid cells (CD4+ and CD8+ alpha-beta T cells, B cells) decreased significantly (FDR ≤10%) during viral infection (Figure 6A, Table S4). These results are in line with recent reports demonstrating increased neutrophil and decreased T-cell counts in patients with COVID-19 (Diao et al., 2020; Liu et al., 2020; Qin et al., 2020). In patients with non-COVID-19 viral infections, we observed significant increase in proportion for myeloid cells (M1 macrophages, CD14 + monocytes, MAST cells) and significant decrease in proportion for lymphoid cells (CD4+ and CD8+ T cells, gamma-delta T cells, B cells) (Figures 6B and S4). Indeed, when considering changes within each data set, M1 macrophages, plasmacytoid dendritic cells, CD14 + monocytes, CD4+ T cells, and total T cells showed change consistently in the same direction across all viral infections including COVID-19 (Figure 6B).

We observed an overall correlation of 0.493 (p = 0.017) for change in cellular proportions in patients with COVID-19 compared to non-COVID-19 viral infections (Figures 6C, Table S4), where all but 6 cell types changed in the same direction, though not all changes were statistically significant. We again observed increased neutrophil and decreased T-cell counts in COVID-19 which is in line with a recent study that compared COVID-19 to the 2009 H1N1^20^. Cell types that increased in COVID-19 relative to non-COVID-19 were CD56^bright^ natural killer (NK) cells, M2 macrophages, and total NK cells. Those that decreased in non-COVID-19 relative to COVID-19 were CD56^dim^ NK cells, memory B cells, and eosinophils. Although change in memory B cells was not statistically significant, the direction of change is expected as patients with non-COVID-19 infection are highly likely to have memory to those viruses, whereas SARS-CoV-2 is a novel coronavirus with no pre-existing memory in the population. Similar findings are reported when the absolute cell counts were measured by flow cytometry in smaller patient populations^20^.

## Discussion

Understanding the pathophysiology of COVID-19 is critical to finding new treatments. Defining the portion of the host response to a novel pandemic virus that is similar to current circulating viral infections is imperative as treatment options are unknown and vaccines non-existent in the early months and thus repurposing drugs that have passed the United States Food and Drug Administration (FDA) safety trials can potentially be informed here. Simultaneously, identifying the biology of the host response that is not similar to circulating viruses may help rank the order with which drugs are repurposed if they do not bolster areas of the immune system succumbing to a virus for which we have no direct immune memory or offer novel targets for new drugs. Here, we take a host response transcriptomics approach using peripheral blood transcriptomics of the immune response to COVID-19 (n=62) compared to 652 non-COVID-19 viral infections spanning 6 viruses. While the vast majority of the host immune response appears to be similar between COVID-19 and other viruses, valuable information under pandemic circumstances, our study highlights some key differences.

The scatterplot of the correlation of the differential expression (relative to HCs) of non-COVID-19 viral infections versus COVID-19 infections illustrates this large proportion of concordance and seemingly small amount of discordance (Figure 4). We found only two genes, *ACO1* and *ATL3*, that were expressed in opposite directions using this method. *ACO1* was over-expressed in COVID-19 versus HC and under-expressed in non-COVID-19 viral infections versus HC, whereas *ATL3* entirely oppositely regulated (Figure 4). Viral replication can occur in infected cells due to a hinderance of the function of the immune cells drawn in to kill infected cells; as well, there are reports of SARS-CoV-1 and SARS-CoV-2 directly infecting immune cells themselves (Gu et al., 2005; Hu et al., 2012; Pontelli et al., 2020). As our data are from whole blood RNA, we cannot conclude precisely which of these mechanisms are responsible for the shifts in these genes' expression; however, prior reports suggest that both genes may be involved in viral replication and immune evasion. *ACO1* is an iron-sulfur protein that regulates ferritin and transferrin. When cellular iron levels are low, the protein binds to iron-responsive elements, which represses translation of ferritin (a protein that stores iron), and simultaneously stabilizes the normally rapidly degraded transferrin receptor mRNA allowing for translation of the receptor and more cellular uptake of iron, which is required for proliferation (Koeller et al., 1989). High levels of ferritin are also indicative of macrophage activation syndrome and have been observed in patients with COVID-19 (Ravelli, 2002; Bataille et al., 2020; Dimopoulos et al., 2020; Giamarellos-Bourboulis et al., 2020). *ATL3* is a member of the integral membrane GTPases. Proper formation of ER tubules is affected by mutations in this gene. Viruses are known to target host organelles to enter a host cell and avoid destruction (Inoue and Tsai, 2013). Lack of *ATL3* results in delayed cargo exit and coat assembly for budding from the ER which is necessary for export of cytokines and chemokines in response to infection; *ATL3* has been linked directly to viral replication in Zika (Monel et al., 2019), although Zika was not studied here.

The power of using COCONUT to combine heterogeneous data sets allowed for a pooled, head-to-head comparison of COVID-19 with non-COVID-19 viral infections, resulting in a 416 gene “COVID-19-specific gene signature” (Table S2B). Interestingly, the differentially expressed genes in this analysis were not enriched for any GO terms. However, there is bias in the annotation of gene ontologies to those that are heavily annotated and studied, often referred to as the “streetlight effect”, so absence of evidence does not denote evidence of absence of coordinated differential response (Haynes et al., 2018a, 2018b; Tomczak et al., 2018). Conversely, this novel combination of genes with these particular effect sizes warrants further investigation as a potential route for novel discoveries (Damelin et al., 2017; Haynes et al., 2018b). Simply reviewing what is known of the immunological function of the top two over- and top two under-expressed genes ranked by Hedges' g ES contextualizes *ACO1* and *ATL3* further with hints of a battle of host versus “novel” pathogen, never encountered by the immune system before. The impact on the function of host immune cells during SARS-CoV-1 and MERS infection is driven by their non-structural proteins and affects the normal production of cytokines compared to that of currently circulating viral infections, such as the repression of interferon proteins/ interferons (IFNs) (Hu et al., 2012; Shah et al., 2020). Recently, Blanco-Melo et al. revealed a dysregulated host response indicative of reduced innate antiviral defenses coupled with excessive cytokine production using cell lines, ferrets, and correlating with two deceased patients with COVID-19 (Blanco-Melo et al., 2020), a phenomenon of novel virus escape mechanisms from host defenses, of which we complement here with even larger numbers of entirely human data.

The most under-expressed gene in the “COVID-19-specific gene signature” is ZC3H13. Knocking this gene down was associated with less RNA methylation N6-methyladenosine (m^6^A), an epigenetic modification commonly found in the viral RNA genomes of hepatitis C virus (HCV), Zika, dengue, yellow fever, and West Nile virus (Wen et al., 2018). Depletion of m^6^A methyltransferases increase HVC viral particle production (Gokhale et al., 2016), which would imply more SARS-CoV-2 viral replication. *ATL3* as mentioned is also included in the “COVID-19-specific gene signature” and is under-expressed in COVID-19. When *ATL3* was knocked down, there was less Zika replication, implying that the under-expression is a host counteractive protective mechanism. The second most under-expressed gene is *AMIGO1*, a gene for which very little is known; however, recent studies on this family of genes (Kuja-Panula et al., 2003) suggest a cell adhesion function. Cell adhesion molecules are a key component of combatting pathogen infections, without which the host may not mount an appropriate response (Etzioni, 1996). Since the “COVID-19-specific gene signature” is derived from direct comparison of COVID-19 versus non-COVID-19 infections, *ZC3H13*, *AMIGO1*, and *ATL3* under-expressed in COVID-19 equates to higher expression in non-COVID-19 infections. One possible interpretation of this under-expression of *ZC3H13* and *AMIGO1* in COVID-19 that could be investigated in future studies is that this novel virus may be inhibiting their expression to escape the host responses that are otherwise functional for previously circulating viral infections.

If indeed the under-expression of *ATL3* in the “COVID-19-specific gene signature” illustrates the tipping scales between the microbe and host and similar to Zika infections, less of this gene expression results in less viral replication; this would imply a protective mechanism rather that host immune evasion. In fact, coronaviruses bud into the ER-Golgi intermediate compartment and in MERS, the C-terminal domain of the M protein was found to contain a trans-Golgi localization signal (Perrier et al., 2019); thus, the role of ATL3 as a way to control viral protein budding presents an exciting avenue for future work. Further to which, the top two over-expressed genes of the “COVID-19-specific gene signature” are coiled-coil and C2 domain containing 2A (*CC2D2A*) and human homeostatic iron regulator or high FE2+ (HFE). CC2D2A plays a critical role in cilia formation (Veleri et al., 2014). Primary cilia microtubule-based sensory organelles that detect mechanical and chemical stimuli are found in almost all cells in the body (Garcia-Gonzalo and Reiter, 2012). Following T-cell receptor signaling, the ciliary trafficking machinery is used to provide spatial control of immune synapses at the interface with the antigen-presenting cell for signaling (Stephen et al., 2018). HFE is a non-classical major histocompatibility (MHC) protein (HLA-H). Mutations disable the ability of this protein to bind β2-microglobulin, a component of the HLA class I molecule, which normally present peptides derived from cytosolic proteins; stagnating presentation of peptide loaded MHC class I molecules at the cell surface (Hollerer et al., 2017). While largely responsible for presenting “endogenous” peptides, during viral infection, this class of HLA is responsible for loading of viral peptides at the ER and trafficking those to the cell surface (Hollerer et al., 2017). HFE is essential in this function as these peptides are presented to T cells or NK cells. The two genes, therefore, are both involved in an effective immune signaling between virally infected cells and the host. HFE is pleotropic in function, and it binds with the transferrin receptor thus reducing affinity for iron loaded transferrin, resulting in less cytoplasmic iron (Taneri et al., 2020). ACO1 is bifunctional as well, a key modulator of mitochondrial iron metabolism, and it is also an essential enzyme in the Krebs cycle (Wood, 2006). Iron metabolism and ATP production are essential for the function of the cell and the proliferation of immune cells. Here, we observe over-expression of *CC2D2A*, *HFE*, and *ACO1* in COVID-19 infections and lower expression in non-COVID-19 previously circulating infections. We interpret this COVID-19 over-expression of genes not intensely involved in non-COVID-19 infections as avenues for future exploration as possible counteractive measures for the novel immune evasion eluded to by the under-expression of *ZC3H13* and *AMIGO1* described above.

All of these genes and their functions need to be molecularly investigated to determine their true role; here, we use them as an illustration of both novel immune evasion and immune defense systems. These measures and counter measures will likely be somewhat different for each patient as they progress through the disease. We see in this cohort gene expression indicative of a beneficial host response whereby HLA class I molecules present viral peptides to the host response for identification and destruction via over-expression of *HFE* and *CC2D2A*, carefully managed iron metabolism and energy production via HFE and ACO1. However, how much over-expression is needed in order to overcome the SARS-CoV-2 virus is not known, and not surprisingly, there are trials underway for the use of pegylated interferon alpha in patients with COVID-19 (2020). This drug is FDA approved for treatment of viral infections such as HCV (Tan et al., 2004; Nile et al., 2020) and showed promise in combination with ribavirin in patients with MERS (Omrani et al., 2014), as one of its mechanisms of action increases MHC class I function (Nile et al., 2020).

Within this signature, we also find genes commonly studied in cancer (e.g. *TP53*, *AKT*, *VEGF*, and *CYCS*). Interestingly, primary cilia house a number of oncogenic molecules including smoothened, *KRAS*, epidermal growth factor receptor, and platelet-derived growth factor receptor (Jenks et al., 2018), and thus, the role in the immune response to COVID-19 would need further investigation. Of the 416 COVID-19-specific genes, we also observe multiple superfamily members of ATP-binding cassette transporters, which facilitate the interaction of multiple immune cells with various classes of lipids. In macrophages and lymphocytes, this alters the plasticity of the cell, dampening the immune response to viral invasion (Hubler and Kennedy, 2016). As well as *ZC3H13*, this gene set includes many other zinc finger proteins. Zinc (Zn^2+^) homeostasis in the cell is tightly regulated as viruses need Zn^2+^ for newly synthesized viral proteins (Lazarczyk and Favre, 2008).

In place of GO terms directly derived from our “COVID-19-specific gene signature”, Figure 5 illustrates the comparison of COVID-19 versus HC to non-COVID-19 versus HC GO terms. We found many downregulated pathways are discordant when comparing to HCs. Within these, a cluster of pathways that are high in COVID-19 and low in non-COVID-19 viral infections involve ribosome-related processes. In SARS-CoV-1 infections, it was determined that viral nsp1 disrupts ribosomal translation of host mRNA while allowing viral translation to continue (Huang et al., 2011). An opposite cluster of pathways that are high in non-COVID-19 viral infections and low in COVID-19 positively regulate cell-cell adhesion, cell activation, leukocyte activation, and immune response-activating cell surface receptor signaling, suggesting a less effective immune response in patients with COVID-19. Of particular interest was the observation that while both diseases had enriched GO terms for type-1 interferon signaling pathways, the significance of this enrichment was lower in COVID-19 (Figure 5). The inspection of the 6 genes above mirrors these discordant pathway findings, supporting the concept of novel biology specific to COVID-19 within a largely similar response to other viruses.

Interestingly, the immune cell proportions are mostly consistent across COVID-19 and non-COVID-19 data sets. Our results are in line with several recent studies that found high neutrophil-lymphocyte ratio in patients with COVID-19 (Diao et al., 2020; Lagunas-Rangel, 2020; Liu et al., 2020; Qin et al., 2020). Expansion of CD56^bright^ NK cells is common in many viral infections, as part of recognizing and killing virally infected cells while orchestrating adaptive immune responses (Vivier et al., 2008). Comparing patients with COVID-19 to HCs shows an increase in NK cells (Figure 6A), largely driven by the CD56^bright^ population. When compared to non-COVID-19 viral infections, the increase in NK cell (via CD56^bright^ NK cell) proportion remains high in the COVID-19 infections. This phenomenon was also directly observed using mass spectrometry to measure cell abundance over time in patients with COVID-19 and when considering factors most explanatory in those that recovered the cells that were the most dynamic included CD56^dim^ NK cells (Sun et al., 2020).

When comparing COVID-19 to non-COVID-19 viral infections, we see M1 macrophage proportions are similar to those of other viral diseases, but the elevated M2 response is discordant. M1 macrophages are pro-inflammatory and kill invaders, whereas M2 macrophages are considered anti-inflammatory and reparative. A large body of work in bacterial sepsis found that individuals with high M1 profiles had increased mortality, whereas those with a more evenly balanced M1/M2 were more likely to survive (Benoit et al., 2008). However, in general, monocytotropic viruses including SARS-CoV-1 have evolved mechanisms to interfere with effective macrophage polarization (Hu et al., 2012), favoring the M2 population for immune evasion. For example, virus-induced macrophage depletion is executed by viruses that carry pro-antiapoptotic proteins, thus initially reducing the number of M1s to skew population to M2 and avoid attack, and then further suppress the production and action of type I IFNs, stunting the progression of M1 macrophage polarization (Laura C Miller, 2015). This shift we see in the proportion of M2 macrophages in COVID-19 versus non-COVID-19 viral infections indicates that this novel pathogen may be executing these immune evasion techniques with a high degree of success. We see that eosinophils and CD56^dim^ natural killer (NK) cells are lower in COVID-19 versus non-COVID-19 infections, which replicated in a system-level study over time using mass cytometry and Olink assays where both cell types increased in abundance from a low level at the acute phase to a normal level in the recovery phase (Rodriguez et al., 2020). As well, decreased B cell and increased M2 macrophage cells were observed in a study of 3939 patients with COVID-19 from China and pose many avenues for novel therapies (Wang et al., 2020).

In conclusion, we here provide bulk RNAseq profiling of peripheral blood in COVID-19 in comparison to HCs which we derived a signature of 2002 genes for investigation of the biology and potentially pathophysiology of this disease, the “COVID-19 signature genes”. We compiled an extensive database of non-COVID-19 viral infections across many platforms, ages, diseases, and locations globally to compare to HCs using metaintegration to derive a set of 635 genes representing the host response to known viral pathogens, the “non-COVID-19 signature genes”. We then used COCONUT to conormalize all of the data and directly compare COVID-19 to non-COVID-19 viral infections resulting in a signature of 416 genes, the “COVID-19-specific gene signature”. We used all of these analyses to identify both the similarities and differences in the underlying host response. While we found that a large proportion of the host response is similar to that of other infections, we also identified key differences in individual genes, pathways, and cellularity that are suggestive of the clinical differences observed in COVID-19. The genes *ACO1* and *ATL3* were identified as an intersect of gene signatures for COVID-19 versus HCs and non-COVID-19 versus HCs, which were further contextualized when considering the top ranking genes of the novel “COVID-19-specific gene signature”*,* suggesting we have illuminated novel biology of the host immune response to a totally novel viral infection, but our findings will need to be replicated in further clinical studies. In summary, COVID-19 gene expression is highly correlated with known viral infection gene expression and has similar shifts in the immune cell proportions known to play a role in viral response but also shows discordant shifts in immune cells that are novel and reflect other recent publications, key information at the onset of a pandemic to leverage our prior and mounting viral infection knowledge. Our computational methods allowed for a head-to-head comparison of COVID-19 and non-COVID-19 viral infection resulting in a novel 416 gene signature, of which many of the genes with the largest Hedges' g ES have well-known immune functions; however, GO terms were not significant suggesting the magnitude and combination of the genes that discriminate the host response to this novel virus can be disseminated to the scientific community at large to investigate whether this novel combination of genes yields any targetable pathophysiology.

### Limitations of the study

Our study has some limitations due to the design of using public data for non-COVID-19 comparison. First, due to the limited nature of clinical studies during a pandemic, we had just 62 patients with COVID-19 compared to >650 with other viral infections, creating class imbalance in their comparison. Second, we did not investigate effects of severity on host response as this was mostly unavailable. It is possible that differences in severity between this COVID-19 cohort and the other viral cohorts was a confounder in our analysis. Third, we analyzed differential expression at single pre-set significance and effect size thresholds. Choosing different thresholds (e.g., thresholds based on 80% statistical power in each analysis) would have identified different sets of differentially expressed genes. We provide Hedges g ES and FDR values for all genes (Table S2A) to enable re-analysis of these genes based on thresholds that others may deem more appropriate. Figure S3 is also provided to show the GO term enrichment results by varying cutoffs.

### Resource availability

#### Lead contact

Timothy E Sweeney, MD, PhD, tsweeney@inflammatix.com, 863 Mitten Rd, Suite 104, Burlingame, CA 94010.

#### Material availability

This study did not generate any new unique reagents and/or materials.

#### Data and code availability

The publicly available studies can be accessed on GEO under their respective study IDs. The COVID-19 cohort is deposited in the Gene Expression Omnibus (GEO) database: GSE152641. Results were generated using R packages COCONUT and MetaIntegrator; both methods have been published and are publicly available R packages. The RNAseq pipeline used to process COVID-19 cohort is described in the methods section.

Additional supplemental items including Transparent Methods are available from Mendeley Data: https://doi.org/10.17632/t4twwtvv7r.1.

## Methods

All methods can be found in the accompanying Transparent Methods supplemental file.

## Supplemental Information

Document S1. Transparent Methods, Figures S1–S4, and Tables S3 and S4

Table S1. RNAseq_tech_data.xlsx, related to Figure 1

Table S2. 9818genes.xlsx, related to Figures 1A, 2A, 4, and 5C
