# Supplementary material for: Transcriptomic similarities and differences in host response between SARS-CoV-2 and other viral infections
Source: iScience. 2020 Dec 16;24(1):101947. doi: 10.1016/j.isci.2020.101947 (PMC7786129; doi:10.1016/j.isci.2020.101947)
Supplement: Document S1. Transparent Methods, Figures S1–S4, and Tables S3 and S4 [file mmc1.pdf]

iScience, Volume *24*

Supplemental Information

Transcriptomic similarities and differences in host response between SARS-CoV-2 and other viral infections

Simone A. Thair, Yudong D. He, Yehudit Hasin-Brumshtein, Suraj Sakaram, Rushika Pandya, Jiaying Toh, David Rawling, Melissa Remmel, Sabrina Coyle, George N. Dalekos, Ioannis Koutsodimitropoulos, Glykeria Vlachogianni, Eleni Gkeka, Eleni Karakike, Georgia Damoraki, Nikolaos Antonakos, Purvesh Khatri, Evangelos J. Giamarellos-Bourboulis, and Timothy E. Sweeney

Supplementary Figure 1. Effect size of DESeq and post COCONUT voom transformed expression data correlate, related to Figure 1.

Supplementary Figure 2. Power Analysis. non-COVID-19 (n=652) versus healthy controls (n=672), related to Table 2 and Figure 2.

Supplementary Figure 3. Heatmap of significance score defined as -log10(P-adjusted) from GO term enrichment analyses. Columns contain 45 gene sets including three gene gets (pos, neg, and all) each from COVID-19 vs HC, non-COVID-19 vs HC, or COVID-19 vs non-COVID-19 comparisons with the chosen cutoff and 3 more or less stringent cutoffs, together with 9 gene sets from Figure 4A (see Supplementary Table 4). Rows represent 252 GO terms filtered from a total 8422, in 10 groups by k-means. Related to Figure 4.

Supplementary Figure 4. Forest plots of cell deconvolution estimates for all studies where estimation was possible (median and interquartile range (IQR)), related to Figure 6.

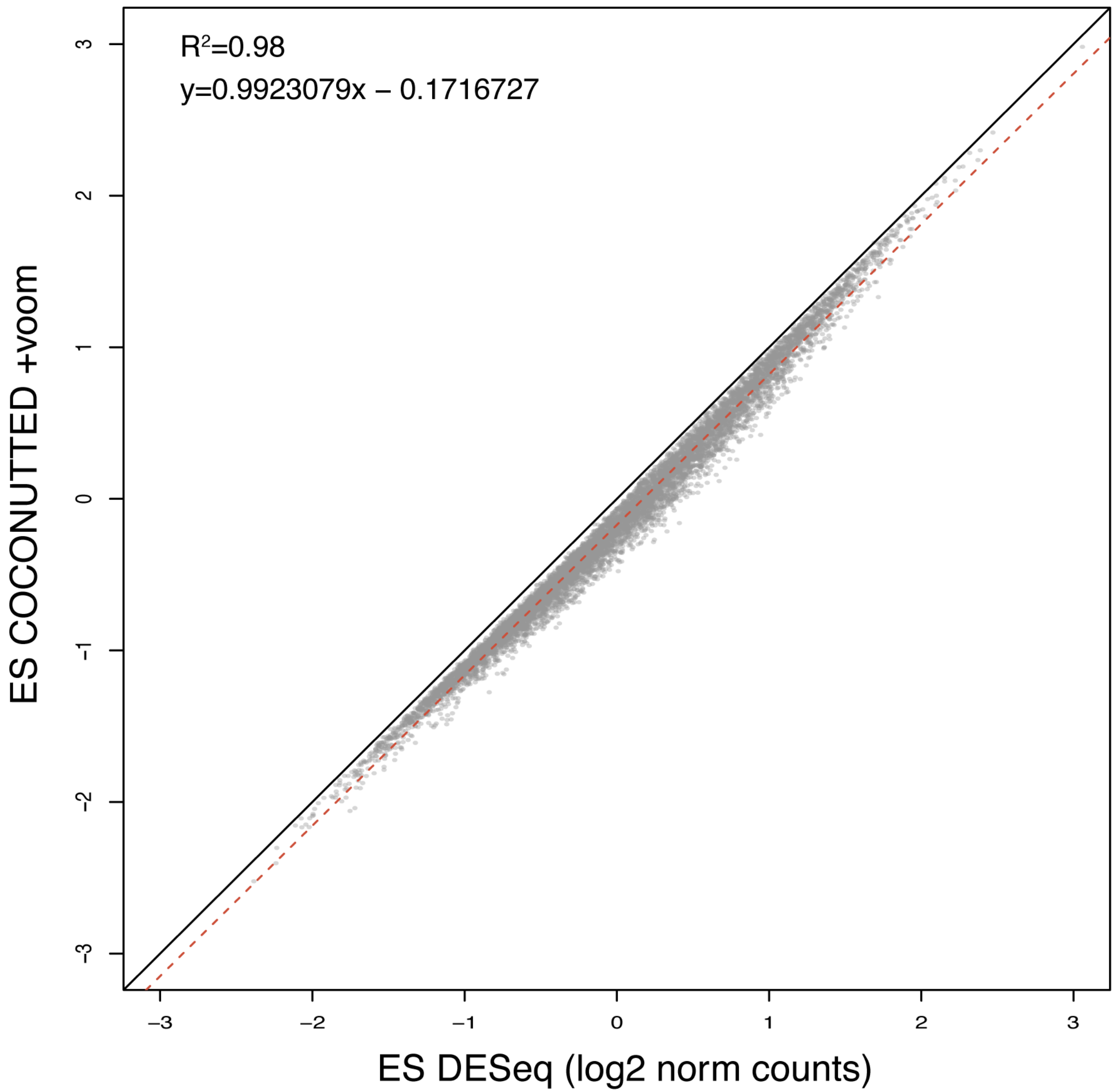

Supplementary Figure 1. Effect size of DESeq and post COCONUT voom transformed expression data correlate.

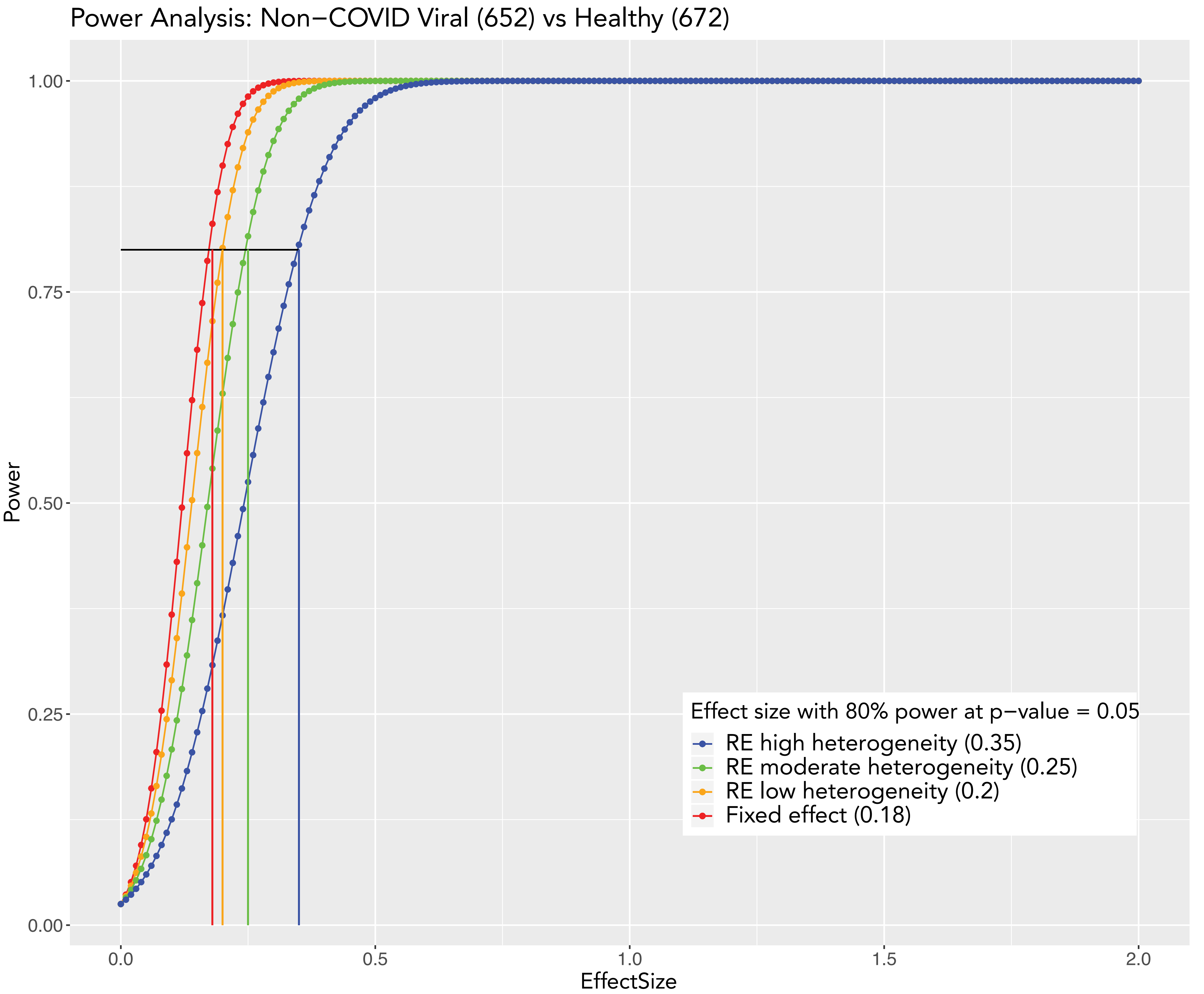

Supplementary Figure 2. Power Analysis. non-COVID-19 (n=652) versus healthy controls (n=672).

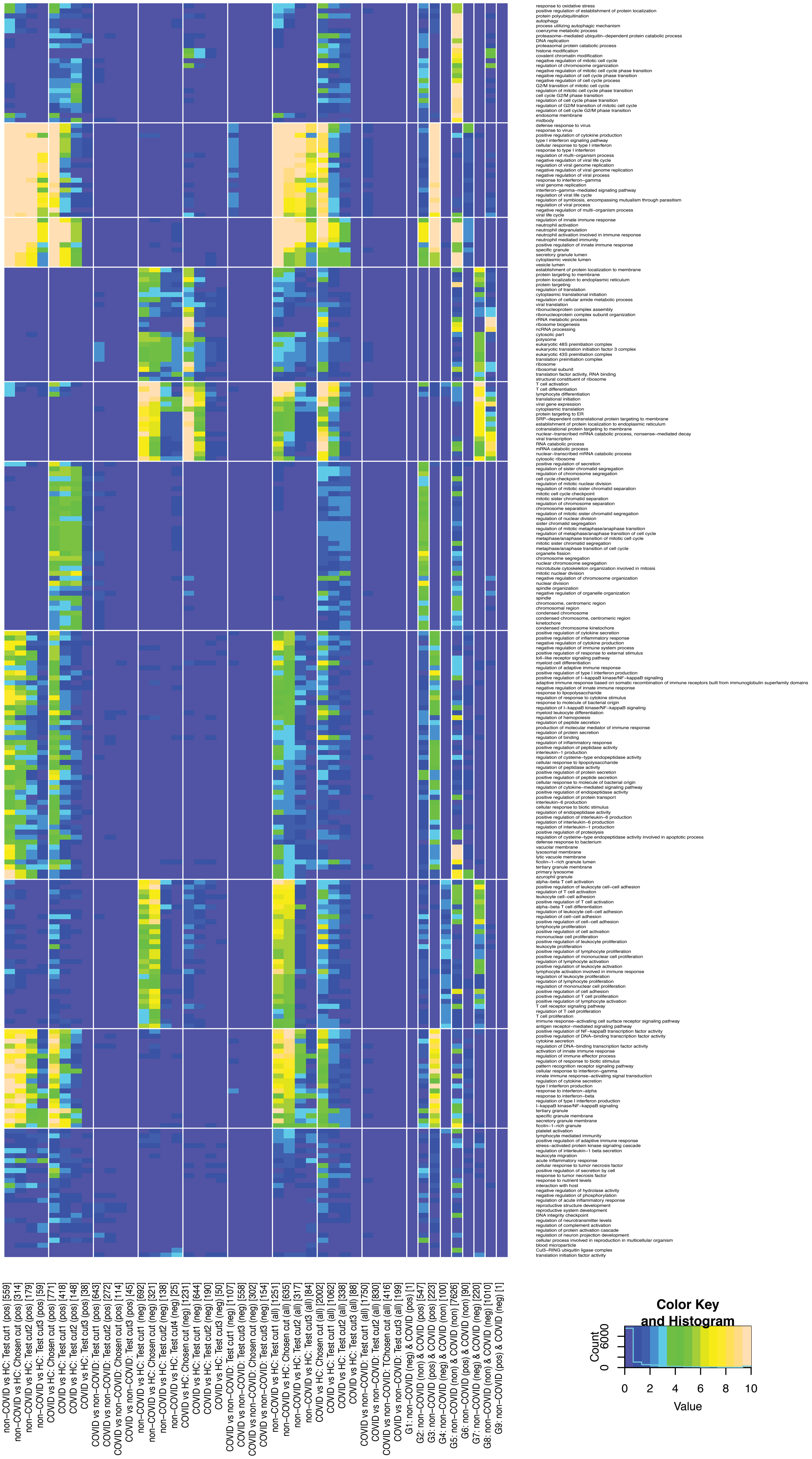

Supplementary Figure 3. Heatmap of significance score defined as -log10(P-adjusted) from GO term enrichment analyses.

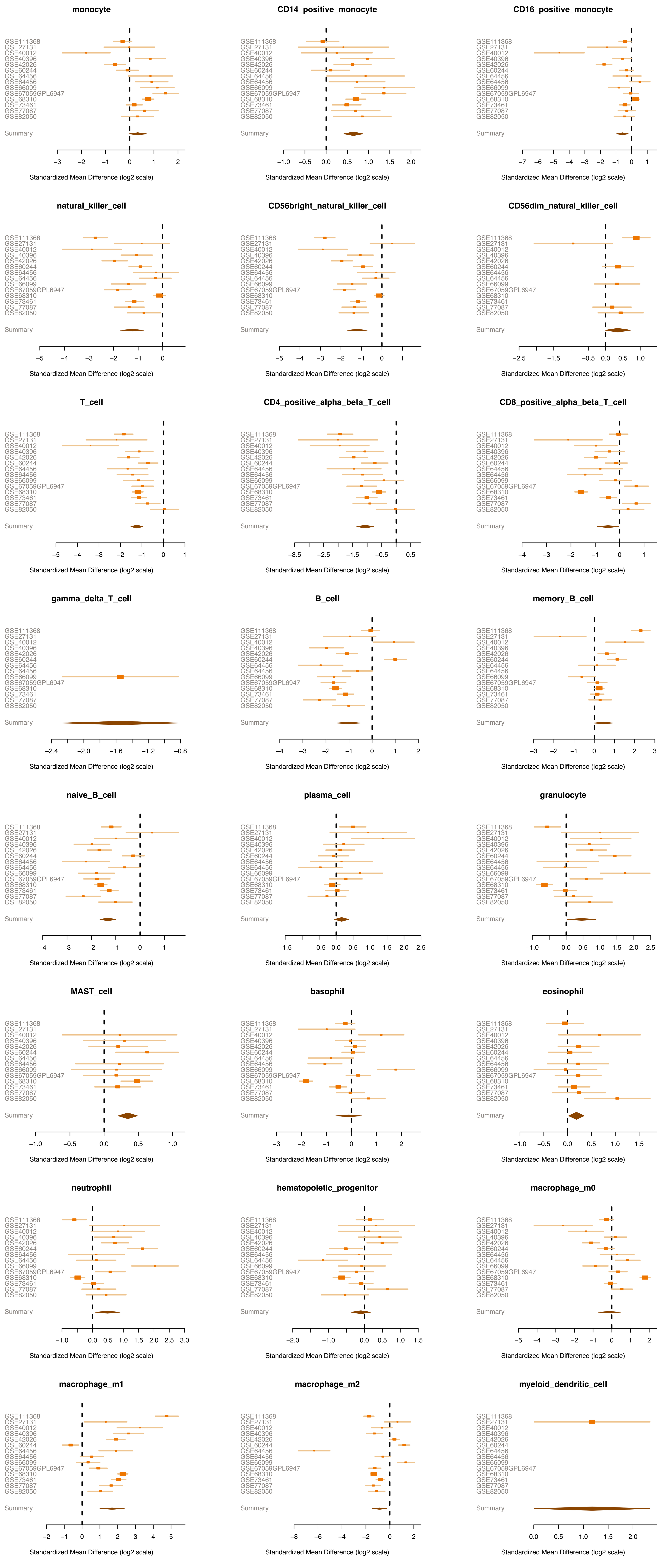

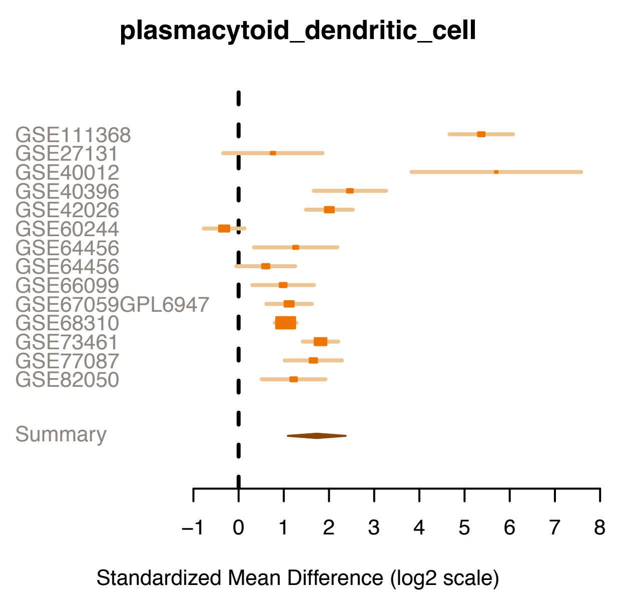

Supplementary Figure 4. Forest plots of cell deconvolution estimates for all studies where estimation was possible (median and interquartile range (IQR))

SupplementaryTable1_RNAseq_tech_data.xlsx, related to Figure 1.
(excel file, external to this pdf)

SupplementaryTable2_9818genes.xlsx, related to Figure 1a, 2a, 4, 5c
(excel file, external to this pdf)

SupplementaryTable3_GeneSet_Summary.pdf, related to Figure 4.

SupplementaryTable4_Immunostates.pdf, related to Figure 6.

		ALL	POS	NEG	Comment
**COVID vs HC comparison**					
Chosen cutoff	IESI>= 1.0 & FDR < 0.05%	2,002	771	1,231	***COVID-19 signature***
Test cutoff	IESI>= 1.2 & FDR < 0.05%	1,062	418	644	
Test cutoff	IESI>= 1.5 & FDR < 0.05%	338	148	190	
Test cutoff	IESI>= 1.8 & FDR < 0.05%	88	38	50	
**non-COVID vs HC comparison**					
Test cutoff	IESI>= 0.8 & FDR < 0.05%	1,251	559	692	
Chosen cutoff	IESI>= 1.0 & FDR < 0.05%	635	314	321	**non-COVID-19 viral signature**
Test cutoff	IESI>= 1.2 & FDR < 0.05%	317	179	138	
Test cutoff	IESI>= 1.5 & FDR < 0.05%	84	59	25	
**COVID vs non-COVID comparison**					
Test cutoff	IESI>= 0.6 & FDR < 0.05%	1,750	643	1,107	
Test cutoff	IESI>= 0.8 & FDR < 0.05%	830	272	558	
Chosen cutoff	IESI>= 1.0 & FDR < 0.05%	416	114	302	***COVID-19-specific genes***
Test cutoff	IESI>= 1.2 & FDR < 0.05%	199	45	154	

Concordant and discordant between COVID vs HC and non-COVID vs HC

Group	Change in COVID vs HC	Change in COVID vs HC	# Genes	Comment
G1	under-expressed	over-expressed	1	under- in non-COVID and over- in COVID: gene *ACO1*
G2	un-changed	over-expressed	547	over-expressed only in COVID
G3	over-expressed	over-expressed	223	concordantly over-expressed in both
G4	under-expressed	un-changed	100	under-expressed only in non-COVID
G5	un-changed	un-changed	7,626	unchanged in both
G6	over-expressed	un-changed	90	over-expressed only in COVID
G7	under-expressed	under-expressed	220	concordantly under-expressed in both
G8	un-changed	under-expressed	1,010	under-expressed only in COVID
G9	over-expressed	under-expressed	1	over- in non-COVD and under- in COVID: gene *ATL3*

SupplementaryTable3_GeneSet_Summary.pdf, related to Figure 4.

Cell_type	effectSize_VvsHC_discovery	effectSizePval_VvsHC_discovery	effectSizeFDR_VvsHC_discovery	effectSize_COVID_RNAseq	effectSizePval_COVID_RNAseq	effectSizeFDR_COVID_RNAseq
hematopoietic_progenitor	-0.100	4.69E-01	5.10E-01	na	na	na
monocyte	0.333	7.55E-02	8.99E-02	0.037356319	0.87652478	0.87652478
CD14_positive_monocyte	0.643	2.49E-08	1.56E-07	0.252959215	0.294325632	0.350616078
CD16_positive_monocyte	-0.592	2.38E-03	4.97E-03	-0.046220905	0.8475549	0.87652478
macrophage_m0	-0.133	6.62E-01	6.78E-01	-0.717995492	0.003630674	0.010438188
macrophage_m1	1.686	1.46E-06	4.44E-06	0.778568269	0.001690821	0.005555553
macrophage_m2	-0.860	6.23E-03	1.20E-02	0.277036473	0.251080557	0.320825156
myeloid_dendritic_cell	1.176	4.82E-02	6.69E-02	0.147697164	0.539442115	0.590817555
plasmacytoid_dendritic_cell	1.729	1.19E-07	5.97E-07	0.247456957	0.304883546	0.350616078
granulocyte	0.461	3.06E-02	4.77E-02	0.862896132	0.000547814	0.002099952
MAST_cell	0.346	1.22E-06	4.34E-06	1.05765339	3.17E-05	0.000243023
basophil	-0.110	6.78E-01	6.78E-01	-0.386601363	0.110580827	0.195643002
eosinophil	0.185	2.55E-02	4.25E-02	-0.64034035	0.009112743	0.02328812
neutrophil	0.486	2.00E-02	3.57E-02	0.88885828	0.000381853	0.002099952
natural_killer_cell	-1.242	3.54E-07	1.47E-06	0.358153451	0.138925266	0.211711188
CD56bright_natural_killer_cell	-1.212	1.60E-06	4.44E-06	0.427431699	0.078226784	0.149934669
CD56dim_natural_killer_cell	0.353	5.79E-02	7.62E-02	-0.28855208	0.232075462	0.313984449
B_cell	-1.017	1.00E-04	2.28E-04	-0.574249089	0.018865325	0.043390247
memory_B_cell	0.452	6.36E-02	7.96E-02	-0.351391802	0.146437667	0.211711188
naive_B_cell	-1.323	1.04E-15	1.30E-14	-0.446724386	0.065922016	0.137836942
plasma_cell	0.162	1.23E-01	1.40E-01	0.350652985	0.147277348	0.211711188
T_cell	-1.234	9.76E-18	2.44E-16	-1.909044387	1.48E-11	2.48E-10
CD4_positive_alpha_beta_T_cell	-1.072	3.02E-13	2.52E-12	-1.887953367	2.15E-11	2.48E-10
CD8_positive_alpha_beta_T_cell	-0.476	3.59E-02	5.28E-02	-0.864275406	0.000537496	0.002099952
gamma_delta_T_cell	-1.546	2.46E-05	6.15E-05	na	na	na

SupplementaryTable4_Immunostates.pdf, related to Figure 6.

Transparent Methods

SAMPLE ACQUISITION AND PROCESSING

COVID-19 samples from Hellenic Sepsis Study Cohort

A total of 76 adult patients with SARS-CoV-2 pneumonia were prospectively enrolled from April 1st to May 4th by department participating in the Hellenic Sepsis Study Group (www.sepsis.gr). Patients were enrolled within the first 24 hours of hospital admission using inclusion criteria of identification of a new lower respiratory tract infection due to COVID-19 defined as the presence of new infiltrate in chest X-ray or chest computed tomography indicative of COVID-19 in a patient without any contact with any healthcare facility the last 90 days. SARS-Cov-2 was detected by positive molecular testing of respiratory secretions. For patients who required mechanical ventilation (MV), blood sampling was performed within the first 24 hours from MV(Giamarellos-Bourboulis *et al.*, 2020). Exclusion criteria were infection by the human immunodeficiency virus, neutropenia, and any previous intake of immunosuppressive medication (corticosteroids, anti-cytokine biologicals, and biological response modifiers). The studies were conducted under approval number 30/20 by the National Ethics Committee of Greece. Written informed consent was provided by patients or by first-degree relatives in cases where patients were unable to consent.

Whole blood was drawn in PAXgene tubes at enrollment along with other standard laboratory parameters. Data collection included demographic information, clinical scores (SOFA, APACHE II), laboratory results, length of stay and clinical outcomes. Patients were followed up daily for 30 days; outcomes were defined as severe respiratory failure (PaO_2/FiO_2 ratio less than 150 requiring MV) or death. PAXgene Blood RNA samples were shipped to Inflammatix for processing.

Healthy control sample sourcing

Blood RNA tubes were prospectively collected from healthy controls (HC) through a commercial vendor (BioIVT) under IRB approval (Western IRB #2016165) using informed consent. Donors were verbally screened to have no inflammation, infection, illness symptoms, (including no fever or antibiotics within 3 days of sampling) or to be immunocompromised. These samples were drawn prior to July 15, 2019, at least 6 months before the first COVID-19 case reported in the US. All samples were tested and negative for HIV, West Nile, Hepatitis B, and Hepatitis C by molecular or antibody-based testing. The age (median and interquartile range (IR) was 36 (29-45.25) and was 70.8% male.

RNA extraction protocol

Prior to processing, samples in PAXgene Blood RNA tubes from 76 COVID-19 patients and 24 healthy controls were removed from -80C to thaw at room temperature for two hours. The samples were then inverted several times to achieve homogeneity, after which 3 mL aliquots were removed for processing. RNA was extracted from these samples using a modified version of the RNeasy Mini Kit (QIAgen) protocol executed on the a QIAcube automated workstation. PAXgene samples comprise of whole blood in PAXgene stabilizing solution. The sample is diluted with PBS, then centrifuged at 3,000 x g to pellet precipitated nucleic acids. Pellets were washed with molecular biology grade water and again pelleted via centrifugation at 3,000 x g. Pelleted material is resuspended in Buffer RLT (QIAgen). Using the automated QIAcube, samples are then subjected to treatment by Proteinase K and gDNA elimination via columns (QIAgen). Flow-through was mixed with isopropanol and passed over a MinElute (QIAgen) spin column. The column was washed with 80% ethanol and purified nucleic acid was eluted in RNase-free water. Purified RNA was heat denatured at 55° C for 5 minutes, then snap-cooled on ice. RNA was quantitated using a Qubit fluorimeter with the Quant-iT RNA Assay kit (Thermo-Fisher). Samples with an RNA integrity number (RIN) below 7 (BioAnalyzer, Agilent) did not proceed to sequencing, resulting in 62 COVID-19 samples and 24 HC samples for sequencing.

RNAseq library preparation

Total RNA samples were depleted of globin RNA using the GLOBINclear kit (Invitrogen) following the procedure described by the manufacturer. Globin-depleted RNA was quantified using the Qubit RNA High Sensitivity kit (Life Technologies) and 10ng of globin-depleted RNA was then used for rRNA depletion and RNAseq library preparation using the SMARTer Stranded Total RNAseq kit v2 Pico Input Mammalian (Takara Bio) following the manufacturer's protocol. RNAseq libraries were then quantified using the Qubit dsDNA High Sensitivity kit (Life Technologies) and their quality and size evaluated by a Fragment Analyzer High Sensitivity Small Fragment kit (Agilent Technologies).

RNA sequencing

A total of 86 RNAseq libraries generated above were pooled and sequenced on an Illumina NovaSeq6000 Sequencing System (Illumina) in a paired-end fashion (2 x 100 cycles). 41 M to 124 M paired-end reads were obtained for each sample obtained for each sample. Fastq files were used as input for RNAseq data processing. Library prep and sequencing were performed at TB-SEQ (Palo Alto, CA).

DATA PROCESSING AND ANALYSIS

RNAseq data processing

Trimming: Quality control (QC) assessment of the reads was done using FastQC(Andrews S, 2018). The adapter sequence and 3 bases on the 3′ end of the reads was trimmed using cutadapt as a commonly used procedure(Martin, 2011).
Alignment: Trimmed reads were mapped to a reference genome index generated based on the human genome, GRCh38, and a transcriptome reference, GENCODE v32 primary assembly gtf(Frankish *et al.*, 2019) with the sjdbOverhang option set to 100 (default), using STAR aligner (v2.7.3a).
Quantitation: Mapped reads were quantified as per Ensembl transcript ID as defined in GENCODE v32 annotation. Reads were summed across Ensembl transcript IDs mapping to Entrez gene IDs in order to compare them with other viral data assayed by microarrays (AnnotationDbi from Bioconductor)(Pagès *et al.*, 2017).
Data Quality: Various QC metrics prior to and post trimming were examined to assess data quality as a standard procedure for RNAseq data. Additionally, the distributions of raw and trimmed counts were assessed and Principal Component Analysis (PCA) with various cutoffs was performed for QC. All 86 samples passed standard QC metrics and the resulting counts matrix (12,142 Entrez genes by 86 samples) was used in subsequent data integration steps (**Supplementary Table 1**).

Normalization and voom transformation of RNAseq counts

Low-expressed genes were filtered using the following cutoff: max counts per million (CPM) less than 5 across all 86 samples. Normalization factors were obtained using edgeR's Trimmed Mean of M values (TMM) method (R package v.3.28.0) (Robinson, McCarthy and Smyth, 2009). The voom method (limma R package v.3.41.18) was then used to transform counts into normalized log2-CPM **(Supplementary Figure 1)**(Ritchie *et al.*, 2015).

Non-COVID-19 viral dataset selection

Transcriptomic data of clinical respiratory infections caused by viruses other than SARS-CoV-2 were surveyed from Gene Expression OmniBus (GEO) and ArrayExpress for inclusion to define a conserved host response signature for non-COVID-19 viral infection. We identified 23 such independent datasets that profiled a total of 1,855 peripheral blood samples (PBMCs or whole blood) from patients (infants, children, or adults) with one of six viral infections (influenza, RSV, HRV, Ebola, Dengue, SARS-CoV-1, but not SARS-CoV-2). Collectively the 23 datasets comprised of 780 samples from healthy controls and 1,075 from patients with a viral infection represent biological,

clinical, and technical heterogeneity observed in the real-world patient population with viral infections.

Non-COVID-19 viral dataset processing

Raw microarray data for each dataset was renormalized (when available) using standardized methods. Affymetrix arrays were renormalized using the robust multichip average (RMA) method. Illumina, Agilent, GE, and other commercial arrays were renormalized via normal-exponential background correction followed by quantile normalization. Data were log2-transformed. Probe to gene (Entrez ID) summarization was performed within each study using the mean signal intensity for probes mapping to a single gene. While there is no consensus in the community, we have used this method across a multitude of studies, being that if more than one probe mapped to a specific gene, probes were summarized with a fixed-effects model because a gene within a sample can have only one expression value(Ramasamy *et al.*, 2008).

COCONUT conormalization of all data sets

Of the 23 non-COVID-19 viral infections datasets, 20 datasets with a total of 879 viral infected patients and 754 HCs met the criteria for conormalization: 1) the dataset must have HCs, and 2) the dataset was obtained on a single-channel microarray platform. The split between discovery and validation is driven first, by computational technicality whereby the 3 datasets that are not COCONUT conormalized automatically are held out for validation. Second, we held out pandemic and non-respiratory viral infections (eg. Dengue) for test of the signature as a type of "global" viral signature. Third, of the remaining respiratory/ non-pandemic we split as per described best practices(Sweeney *et al.*, 2017) in concert with similar distribution of the types of viruses in discovery and validation.

Integrated with the voom-transformed RNAseq dataset for COVID-19, they were conormalized together using COCONUT (R package v. 1.0.2) (Sweeney, Wong and Khatri, 2016). COCONUT uses COMBAT empiric-Bayes conormalization on healthy controls to derive correction factors for diseased patients. The technique integrates datasets such that (i) no bias is introduced to the diseased samples, (ii) there is no change to the distribution of a gene within a study, and (iii) each gene shares the same distribution across healthy controls between studies after normalization. This COCONUT conormalized expression data comprising of a total of 941 (COVID-19 and non-COVID-19) viral patients and 778 HCs across 9,818 genes common across 11 platforms were used as input data to perform the following multicohort and integrated analyses.

COVID-19 versus healthy control comparison

Hedges' g effect size (ES)(Hedges and Olkin, 1985) for each gene was calculated for COVID-19 (62) versus HC (24) two-group comparison test from the COCONUT conormalized output. Hedges' g is the difference between groups as a proportion of variability in the groups and is calculated as:

$$g = \frac{(\bar{X}_1 - \bar{X}_2)}{S_{pooled}}$$

Whereby $\bar{X}_1$ and $\bar{X}_2$ are sample means in two groups. This is divided by the "within-groups" standard deviation which is S_{pooled}

$$S_{pooled} = \sqrt{\frac{(n_1 - 1)S_1^2 + (n_2 - 1)S_2^2}{n_1 + n_2 - 2}}$$

Where n_1 and n_2 are the sample sizes in the two groups, and S_1 and S_2 are the respective standard deviations. The estimation of effect size for smaller studies is corrected with J.

$$J = 1 - \frac{3}{4df - 1}$$

P-value was calculated using a student's t-test and adjusted using the Benjamini-Hochberg method to obtain the False Discovery Rate (FDR). Hedges' g ES threshold of ≥ 1 or ≤ -1 in combination with FDR threshold of ≤ 0.05% was used to identify genes whose expressions are over- or under-expressed in COVID-19 infected patients than in the mean value of HCs **(see Detailed Meta-Analysis section below for an expanded description).**

Non-COVID-19 viral versus healthy controls comparison

14 datasets composed of 1,324 whole blood and PBMC samples were chosen for the discovery cohort, of which 652 were from respiratory viral infected patients (viral) and 672 samples were from HCs patients. As a multi-cohort analysis with conormalized data as input, we utilized a well-established MetaIntegrator (version 2.1.1)(Haynes *et al.*, 2017). Briefly, Hedges' g ES was computed for each gene within a study between viral and HC. ESs for genes across studies was summarized using the DerSimonian & Laird random-effects model, where each ES is weighted by the inverse of the variance in that study(DerSimonian and Laird, 2015) **(Supplementary Methods).** We used an ES threshold ≥ 1 or ≤ -1 with FDR ≤ 0.05% to identify signature genes (**Supplementary Table 2**).

Validation of non-COVID-19 viral infection signature

The signature genes identified based on 14 discovery datasets were evaluated for prediction of viral infections from HC with a score calculated for each sample using the following formula:

$$viral\ score = zscore\big(GeoMean(pos) - GeoMean(neg)\big)$$

The score is a rescaled difference between geometric means of positive (over-expressed) genes and negative (under-expressed) genes. Receiver-operating characteristics (ROC) plots are generated for held out validation datasets and the Area Under the ROC (AUC or AUROC) is used as a performance metric.

For validation of the non-COVID-19 viral signature, we compiled 9 datasets comprised of 6 held out from the COCONUT expression data, plus 3 normalized as per platform requirements without COCONUT (**Table 3**). We then tested this signature first using 4 datasets comprising of 178 respiratory viral infection samples and 58 HCs (236 total) (**Table 3**). We then further validated this signature in 5 datasets of other viral etiology (245 viral and 50 HC, 295 total) **(Table 3).**

COVID-19 versus non-COVID-19 viral Comparison

Hedges' g ES was calculated for each gene in a COVID-19 (62) and non-COVID-19 viral (652) two-group comparison test from the COCONUT conormalized expression data. P-value was calculated using a Welch's t-test assuming unequal variance and sample sizes and adjusted using the Benjamini-Hochberg(Benjamini and Hochberg, 1995) method to obtain the False Discovery Rate (FDR). ES threshold ≥ 1 or ≤ -1 in combination with FDR threshold of $\leq 0.05\%$ was used to identify signature genes.

PATHWAY AND IMMUNOSTATES ANALYSIS

Pathway Analysis

Each over- or under-expressed gene set from comparisons between COVID-19 vs HC, non-COVID-19 viral infection vs HC, and COVID-19 vs non-COVID-19 viral infection was subjected to a pathway analysis with Gene Set Enrichment Analysis (GSEA)(Subramanian *et al.*, 2005). We tested significance of over-representation of genes in each of the pathways reflected in Gene Ontology (GO) including biological process (BP), molecular function (MF), and cellular compartment (CC). The human transcriptome reference is used as background and the p-values from the hyper-geometric test were adjusted using the Benjamini-Hochberg method(Benjamini and Hochberg, 1995). Top-ranked pathways common between COVID-19 and non-COVID-19, and specific separately to COVID-19 or non-COVID-19 viral infections were selected.

ImmunoStates Analysis

A statistical deconvolution method was used to estimate the percentage of 25 immune cell types in the peripheral blood transcriptome data (Bongen *et al.*, 2018; Vallania *et al.*,

2018). Statistical deconvolution estimates the percentage of various cell types present in a blood transcriptome profile. It uses a set of pre-defined genes that represent cell types of interest, called a basis matrix, and a variant of linear regression to make estimates. Previously, it was demonstrated that different methods produce highly correlated estimates of cellular proportions once basis matrix is fixed(Vallania *et al.*, 2018). Here, immunoStates (MetaIntegrator) was used as a basis matrix because it has been shown to reduce the effect of the biological and technical heterogeneity in transcriptome data on statistical deconvolution and identify robust changes in immune cell proportions(Bongen *et al.*, 2018; Roy Chowdhury *et al.*, 2018; Vallania *et al.*, 2018; Scott *et al.*, 2019). The 14 non-COVID-19 viral discovery datasets and the COVID-19 dataset were deconvolved separately, then change in proportion of a given cell type between healthy controls and the infected patients of each dataset was estimated.

DETAILED META-INTEGRATION STATISTICAL METHODS

The use of Hedges' g (Hedges, 1981; Hedges and Olkin, 1985) effect size (ES) stems from a need for the standardized mean difference to transform all effect sizes to a common metric, and thus enables us to include different outcome measures in the same synthesis. To estimate the standardized mean difference (g) can be calculated as

$$g = \frac{(\bar{X}_1 - \bar{X}_2)}{S_{pooled}}$$

Whereby $\bar{X}_1$ and $\bar{X}_2$ are sample means in two groups.
This is divided by the "within- groups" standard deviation which is S_{pooled}

$$S_{pooled} = \sqrt{\frac{(n_1 - 1)S_1^2 + (n_2 - 1)S_2^2}{n_1 + n_2 - 2}}$$

Where n_1 and n_2 are the sample sizes in the two groups, and S_1 and S_2 are the respective standard deviations.
The estimation of effect size for smaller studies is corrected with J.
By pooling the two estimates of the standard deviation results in a more accurate estimate of their common by including the variance as well as the means.
Hedges (1981) determined that there is a small bias in d (namely Cohen's d) (Cohen, 1988) in small sample sizes, resulting in the addition of a correction factor J

$$J = 1 - \frac{3}{4df - 1}$$

Whereby the df used to estimate S_{pooled} in two independent groups for example would be
$n_1 + n_2 - 2$. J is always less than one and as samples sizes increase, J becomes closer to 1 and thusly impacts smaller sample sizes appropriately without major adjustment in large sample sizes. This is ideal in meta-analysis where the sizes of the studies available often vary.

Thus a gene's Hedges' g effect size represents the difference between groups transformed as a common metric taking into account the proportion of variability in the groups.

This dovetails with the MetaIntegration methods we have developed (MetaIntegrator R package v. 2.1.1)(Haynes *et al.*, 2017), whereby to then pool these effect sizes across datasets, the summary effect size g_s is calculated using a random effects model:

$$g_s = \frac{\sum_i^n W_i g_i}{\sum_i^n W_i}$$

Where n is the number of datasets, W_i is a weight equal to $\frac{1}{(V_i + T^2)}$, where V_i is the variance of that gene within a given dataset I and T^2 is the inter-dataset variation estimated using the DerSimonian- Liard method(DerSimonian and Laird, 2015), determined to be optimal for our methods but may be further investigated by each individual research group (Sweeney *et al.*, 2017).

Standard error for the summary effect size is derived with

$$SE_{g_s} = \sqrt{\frac{1}{\sum_i^n W_i}}$$

From which a p-value is calculated and corrected for using the Benjamini-Hochberg(Benjamini and Hochberg, 1995) false discovery rate (FDR) correction for multiple hypotheses. Fisher's method is used for combining p-values across the studies as the log sum of

$$F_{up} = -2\sum_{i}^{n} \log{(p_i)}$$

For up-regulated genes and again for down regulated genes, these are again corrected with the Benjamini- Hochberg method.

Genes can then be filtered based on ES, ES FDR, Fisher's FDR and if desired, MetaIntegrator allows for the inclusion of genes that meet these criteria in a leave one dataset out analyses and can further be queried for Cochrane's Q value for evaluating heterogeneity of effect size estimates between studies:

$$Q = \sum_{i}^{n} W_i\,(g_i - g_i)^2$$

Allowing for the user to increase the number of genes to pursue perhaps in the case of biological interest or restrict the number of genes when searching for predictive signatures in a disease type (Haynes *et al.,* 2017; Sweeney *et al.,* 2017).

Andrews S (2018) 'FastQC A Quality control tool for high throughput sequence data', *Babraham Bioinfo*.

Benjamini, Y. and Hochberg, Y. (1995) 'Controlling the False Discovery Rate: A Practical and Powerful Approach to Multiple Testing', *Journal of the Royal Statistical Society: Series B (Methodological)*. doi: 10.1111/j.2517-6161.1995.tb02031.x.

Bongen, E. *et al.* (2018) 'KLRD1-expressing natural killer cells predict influenza susceptibility', *Genome Medicine*. doi: 10.1186/s13073-018-0554-1.

Cohen, J. (1988) *Statistical Power Analysis for the Behavioural Science (2nd Edition), Statistical Power Anaylsis for the Behavioral Sciences*.

DerSimonian, R. and Laird, N. (2015) 'Meta-analysis in clinical trials revisited', *Contemporary Clinical Trials*. doi: 10.1016/j.cct.2015.09.002.

Frankish, A. *et al.* (2019) 'GENCODE reference annotation for the human and mouse genomes', *Nucleic Acids Research*. doi: 10.1093/nar/gky955.

Giamarellos-Bourboulis, E. J. *et al.* (2020) 'Complex Immune Dysregulation in COVID-19 Patients with Severe Respiratory Failure', *Cell Host and Microbe*. doi: 10.1016/j.chom.2020.04.009.

Haynes, W. A. *et al.* (2017) 'Empowering multi-cohort gene expression analysis to increase reproducibility', in *Pacific Symposium on Biocomputing*. doi: 10.1142/9789813207813_0015.

Hedges, L. V. (1981) 'Distribution Theory for Glass's Estimator of Effect size and Related Estimators', *Journal of Educational Statistics*. doi: 10.3102/10769986006002107.

Hedges, L. V and Olkin, I. (1985) *Statistical Methodblogy in Meta-Analysis., Statistical Methodblogy in Meta-Analysis*.

Martin, M. (2011) 'Cutadapt removes adapter sequences from high-throughput sequencing reads', *EMBnet.journal*. doi: 10.14806/ej.17.1.200.

Pagès, H. *et al.* (2017) 'Package "AnnotationDbi"', *Bioconductor Package Maintainer*.

Ramasamy, A. *et al.* (2008) 'Key issues in conducting a meta-analysis of gene expression microarray datasets', *PLoS Medicine*. doi: 10.1371/journal.pmed.0050184.
Ritchie, M. E. *et al.* (2015) 'Limma powers differential expression analyses for RNA-sequencing and microarray studies', *Nucleic Acids Research*. doi: 10.1093/nar/gkv007.
Robinson, M. D., McCarthy, D. J. and Smyth, G. K. (2009) 'edgeR: A Bioconductor package for differential expression analysis of digital gene expression data', *Bioinformatics*. doi: 10.1093/bioinformatics/btp616.
Roy Chowdhury, R. *et al.* (2018) 'A multi-cohort study of the immune factors associated with M. tuberculosis infection outcomes', *Nature*. doi: 10.1038/s41586-018-0439-x.
Scott, M. K. D. *et al.* (2019) 'Increased monocyte count as a cellular biomarker for poor outcomes in fibrotic diseases: a retrospective, multicentre cohort study', *The Lancet Respiratory Medicine*. doi: 10.1016/S2213-2600(18)30508-3.
Subramanian, A. *et al.* (2005) 'Gene set enrichment analysis: A knowledge-based approach for interpreting genome-wide expression profiles', *Proceedings of the National Academy of Sciences of the United States of America*. doi: 10.1073/pnas.0506580102.
Sweeney, T. E. *et al.* (2017) 'Methods to increase reproducibility in differential gene expression via meta-analysis', *Nucleic Acids Research*. doi: 10.1093/nar/gkw797.
Sweeney, T. E., Wong, H. R. and Khatri, P. (2016) 'Robust classification of bacterial and viral infections via integrated host gene expression diagnostics', *Science Translational Medicine*. doi: 10.1126/scitranslmed.aaf7165.
Vallania, F. *et al.* (2018) 'Leveraging heterogeneity across multiple datasets increases cell-mixture deconvolution accuracy and reduces biological and technical biases', *Nature Communications*. doi: 10.1038/s41467-018-07242-6.
